# Tracking Key Industrial Sectors for CO_2_ Mitigation through the Driving Effects: An Attribution Analysis

**DOI:** 10.3390/ijerph192114561

**Published:** 2022-11-07

**Authors:** Xian’en Wang, Tingyu Hu, Junnian Song, Haiyan Duan

**Affiliations:** 1 Key Laboratory of Groundwater Resources and Environment, Ministry of Education, Jilin University, Changchun 130021, China; 2 College of New Energy and Environment, Jilin University, Changchun 130012, China; 3 Jilin Provincial Key Laboratory of Water Resources and Environment, Jilin University, Changchun 130021, China

**Keywords:** CO_2_ emissions, industry, attribution analysis, key sectors, emission peak

## Abstract

The heavy pressure to improve CO_2_ emission control in industry requires the identification of key sub-sectors and the clarification of how they mitigate CO_2_ emissions through various actions. Focusing on 30 Chinese provincial regions, this study quantifies the contribution of each industrial sector to regional CO_2_ mitigation by combining the logarithmic mean Divisia index with attribution analysis and extract the key sectors of CO_2_ mitigation for each region. Results indicate that during 2010–2019, significant emission reduction was achieved through energy intensity (74%) in Beijing, while emission reductions were attained through industrial structure changes for Anhui (50%), Henan (45%), and Chongqing (45%). The contribution to emission reduction through energy structures is not significant. The production and supply of power and heat (PSPH) is a central factor in CO_2_ mitigation through all three inhibitive factors. *Petroleum processing and coking* (*PPC*) generally contributes to emission reduction through energy structures, while the *smelting and pressing of ferrous metals* (*SPMF*) through changes in industrial structures and energy intensity. *PSPH* and *SPMF,* in most regions, have not achieved the emission peak. Except in the case of *coal mining and dressing* (*CMD*), CO_2_ emissions in other key sectors have almost been decoupled from industrial development. *CMD* effectively promotes CO_2_ mitigation in Anhui, Henan, and Hunan, with larger contribution of *PPC* in Tianjin, Xinjiang, Heilongjiang, and that of *smelting and pressing of nonferrous metals* in Yunnan and Guangxi. The findings help to better identify key sectors across regions that can mitigate CO_2_ emissions, while analyzing the critical emission characteristics of these sectors, which can provide references to formulating region- and sector-specific CO_2_ mitigation measures for regions at different levels of development.

## 1. Introduction

Facing the increasingly severe threat of global climate change, active measures are being invoked around the world to meet this realistic challenge of reducing CO_2_ emissions [1,2,3]. With rapid global industrialization, industry has become the main source of CO_2_ emissions. As the International Energy Agency (IEA) reported, existing industrial plants and coal-fired power plants around the world will emit about 600 billion tons of CO_2_ over the next five years [4]. In the context of meeting more stringent climate targets, the control of industrial CO_2_ emissions should be placed at a prioritized position.

At present, studies on industrial CO_2_ emissions have mainly focused on the influencing factors of emissions and the prediction of emission peak. A great number of studies employed the methods, such as logarithmic mean Divisia index (LMDI) [5,6], stochastic impacts by regression on population, affluence, and technology (STIRPAT) [7,8] and structural decomposition analysis (SDA) [9,10] to identify the influencing factors of industrial CO_2_ emissions. The promotive and inhibitive drivers of emissions were explored at the national [11,12], provincial [13,14], regional [15,16], and sectoral levels [17,18]. The main factors identified included: economic growth, industrial structure, energy structure, energy intensity, population, urbanization, and so on [19,20,21,22]. Economic growth is considered as a primary contributor to increasing emissions, while energy intensity significantly inhibits emissions [23,24]. In addition, other factors, such as energy-saving technology, production efficiency, and research and development (R&D) efficiency, have also been gradually incorporated by researchers into the critical factors [25,26].

Recently, CO_2_ emission peak has been frequently described and analyzed in the prediction of emissions in the scenarios of future economic development. Most studies believe that the CO_2_ emissions of China could reach the peak before 2030 [27,28,29], and industry plays a leading role in controlling emissions, as well as in achieving an earlier peak [30]. Industrial CO_2_ emissions have a potential to peak by 2025 through policies such as optimizing the industrial structure and eliminating overcapacity [31,32]. More subdivided industrial sectors, such as nonferrous metals industry [33], chemical industry [34], power generation industry [35], and steel and cement industries [36] have been estimated to reach the peak of their emissions in different years, and show diverse emission reduction potentials. In numerous scenario analyses, the growth and differences of energy consumption and CO_2_ emissions were predicted and compared under different settings of evolution of socioeconomic situations, for which corresponding policy suggestions were put forward. Ref. [37] believed that under the current policy scenario, the emissions of the power industry in China cannot reach their peak before 2030, the findings of which align with that of Meng et al. [38] based on the prediction of power industry’s emissions in a variety of scenarios. Li et al. [39] assessed the CO_2_ reduction potential of the iron and steel industries in China in six scenarios, providing a feasible path for the industries to achieve the emission reduction goal by 2030.

Reducing industrial CO_2_ emissions is the key to reaching the peak of the whole country’s emissions at an earlier date, which requires a combination of technological innovation and policy guidance. Specific to various sectors, clean coal technology can provoke cleaner power generation development by improving the efficiency of coal utilization for thermal power generation [40]. Waste heat energy recovery technology, hydrogen-based steelmaking, and iron-ore electrolysis technologies are effective ways for sustainable green iron and steel manufacturing [41]. Technology is only one part of the blueprint for emission control, and putting in place the right policies can provide incentives for technology deployment and accelerate emission reduction. For example, the carbon emission trading scheme can stimulate effective responses across all channels, which is an effective approach to achieving emission reduction targets at a sub-national scale [42,43]. The effects of carbon pricing on critical earth system processes have also been explored, as well as the impact on CO_2_ emissions [44]. Given the continuous transformation of development mode and the adjustment of industrial structures, the emission characteristics of different subdivided industrial sectors are diverse. Technological innovation and policy formulation targeting the key sectors are the guarantee of achieving the emission peak as soon as possible. Thus, identifying which sectors deserve prioritized attention is the prerequisite for addressing these issues.

Based on the multiplicative decomposition of LMDI, the attribution analysis traces the change of decomposition factors in various sub-sectors, and, thus, quantifies the contribution of various sectors to variations in CO_2_ emissions, which provides a methodological support for the identification of the “key sectors”. At present, this method has been used to identify the key sectors in terms of energy intensity [45,46], CO_2_ intensity [47,48], air pollutant emissions [49], etc. When evaluating the emission performance of regional energy-related activities, for example in power generation, regional attribution analysis was also used to highlight regional differences of emission reduction [50]. Attribution analysis can ascribe the contributions of the driving factors to some individual components, such as sub-sectors or sub-regions, laying a foundation for the formulation of differentiated policies for different sectors or regions.

The ability of the industry to reach the peak of CO_2_ emissions ahead of schedule is the key to China’s commitment to address climate change. Some problems remain to be further explored, beyond a number of extant studies on industrial CO_2_ emissions. First, the urgency to mitigate emissions should be differentiated among industrial sectors. Identifying the key sectors in terms of larger contribution to CO_2_ mitigation is the basis for an orderly emission reduction route. However, the identification of the key sectors in existing studies mainly relies on qualitative analysis or direct observation based on numerical results, lacking a methodological basis. In addition, industrial energy consumption and technological level vary considerably across the provincial regions as a result of different levels of industrial development and relevant policies. This leads to differences in the key sectors across regions, as well as in the emission characteristics of these sectors, including the decoupling from industrial development and the emission peaking status (having peaked or not). The inadequacy in clarification of the above hinders the formulation of pertinent mitigation measures for industrial CO_2_ emissions.

In light of the above, the CO_2_ emissions of industry in China’s 30 provincial regions are sorted into contributions from six major influencing factors through the LMDI method, and those inhibiting emissions in each region are identified. Then, the contributions of industrial sectors to variations in the inhibitive effects are quantified by the attribution analysis to extract the key sectors that significantly contribute to CO_2_ mitigation. Finally, the emission characteristics of the key sectors in terms of the decoupling from industrial development and peaking status in each region are analyzed. The novelty of this paper lies in (1) tracking the key industrial sectors through the inhibitive driving effects on CO_2_ mitigation in each provincial region in China; (2) inventorying the critical emission characteristics of the key sectors in each region to facilitate the formulation of differentiated emission control measures.

## 2. Methods

### 2.1. Divisia Decomposition Analysis

The LMDI decomposition method has two forms: addition and multiplication [51]. Compared with other index decomposition methods, LMDI has the advantages of wider application scope and easier interpretation of results [52]. In order to conduct the attribution analysis, the LMDI multiplication decomposition method proposed by Choi and Ang [53] is adopted here. The CO_2_ emissions are decomposed into the Kaya identity [54] expressed as:

(1) $$C=\sum_{i}\sum_{j}C_{ij}=\frac{C_{ij}}{E_{ij}}\times \frac{E_{ij}}{E_{i}}\times \frac{E_{i}}{Q_{i}}\times \frac{Q_{i}}{Q}\times \frac{Q}{P}\times P$$

where *C* is total CO_2_ emissions of the industry; *i* denotes an industrial sector and *j* denotes an energy type; *E* is energy consumption; *Q_i_* is the industrial output of sector *i* and *Q* is total industrial output of a region; *P* is population of a region; *C_ij_*/*E_ij_* denotes the CO_2_ emission coefficient of energy *j* in sector *i* (*ED_ij_*); *E_ij_*/*E_i_* denotes the energy structure of sector *i* (*ES_ij_*); *E_i_*/*Q_i_* denotes the energy intensity of sector *i* (*EI_i_*); *Q_i_*/*Q* denotes industrial structure (*IS_i_*)*; Q*/*P* denotes per capita industrial development level (*IO*).

The variations in total CO_2_ emission over the period [*t* − 1, *t*] can be calculated as follows:

(2) $$\frac{C_{t}}{C_{t-1}}=D_{ED}^{t-1,t}\times D_{ES}^{t-1,t}\times D_{EI}^{t-1,t}\times D_{IS}^{t-1,t}\times D_{IO}^{t-1,t}\times D_{P}^{t-1,t}$$

where $D_{ED}^{t-1,t}$, $D_{ES}^{t-1,t}$, $D_{EI}^{t-1,t}$, $D_{IS}^{t-1,t}$, $D_{IO}^{t-1,t}$ and $D_{P}^{t-1,t}$ denote the variations in CO_2_ emissions induced by emission coefficient, energy structure, energy intensity, industrial structure, per capita industrial development level, and population, respectively. The variables in Equation (2) are calculated as follows:

(3) $$D_{ED}^{t,t-1}=\exp(\sum_{i=1}^{I}\sum_{j=1}^{J}\omega_{ij}^{s-v}\ln(\frac{ED_{ij,t}}{ED_{ij,t-1}}))$$

(4) $$D_{ES}^{t,t-1}=\exp(\sum_{i=1}^{I}\sum_{j=1}^{J}\omega_{ij}^{s-v}\ln(\frac{ES_{ij,t}}{ES_{ij,t-1}}))$$

(5) $$D_{EI}^{t,t-1}=\exp(\sum_{i=1}^{I}\sum_{j=1}^{J}\omega_{ij}^{s-v}\ln(\frac{EI_{ij,t}}{EI_{ij,t-1}}))$$

(6) $$D_{IS}^{t,t-1}=\exp(\sum_{i=1}^{I}\sum_{j=1}^{J}\omega_{ij}^{s-v}\ln(\frac{IS_{ij,t}}{IS_{ij,t-1}}))$$

(7) $$D_{IO}^{t,t-1}=\exp(\sum_{i=1}^{I}\sum_{j=1}^{J}\omega_{ij}^{s-v}\ln(\frac{IO_{ij,t}}{IO_{ij,t-1}}))$$

(8) $$D_{P}^{t,t-1}=\exp(\sum_{i=1}^{I}\sum_{j=1}^{J}\omega_{ij}^{s-v}\ln(\frac{P_{ij,t}}{P_{ij,t-1}}))$$

where

(9) $$\omega_{ij}^{s-v}=\frac{L(C_{ij,t}/C_{t},C_{ij,t-1}/C_{t-1})}{\sum_{i=1}^{I}\sum_{j=1}^{J}L(C_{ij,t}/C_{t},C_{ij,t-1}/C_{t-1})}$$

and *L*(*A*, *B*) = (*A* − *B*)/(ln*A* − ln*B*) represents the logarithmic mean function.

The multi-period divisia decomposition expresses total emission variations over the period [0, *T*], which can be expressed as:

(10) $$\frac{C_{T}}{C_{0}}=\prod_{t=1}^{T}\frac{C_{t}}{C_{t-1}}=\prod_{t=1}^{T}(D_{ED}^{t,t-1}\times D_{ES}^{t,t-1}\times D_{EI}^{t,t-1}\times D_{IS}^{t,t-1}\times D_{IO}^{t,t-1}\times D_{P}^{t,t-1})\;=D_{ED}^{0,T}\times D_{ES}^{0,T}\times D_{EI}^{0,T}\times D_{IS}^{0,T}\times D_{IO}^{0,T}\times D_{P}^{0,T}$$

where $D_{ED}^{0,T}$, $D_{ES}^{0,T}$, $D_{EI}^{0,T}$, $D_{IS}^{0,T}$, $D_{IO}^{0,T}$, and $D_{P}^{0\;,T}$ denote the corresponding cumulative products of single-period decomposed variables.

### 2.2. Attribution Analysis

Attribution analysis is used to further explore the contribution of individual components to the effects of influencing factors [53]. Based on the results of the LMDI multiplicative decomposition, this method can attribute the variations in decomposition factors’ effects to all terminal sectors. For example, the single-period attribution results of energy intensity can be expressed as:

(11) $$\frac{EI_{t}}{EI_{t-1}}-1=\sum_{i=1}^{I}\sum_{j=1}^{J}r_{ij}(\frac{EI_{i,t}}{EI_{i,t-1}}-1)\;r_{ij}=\frac{\frac{\omega_{ij}^{s-v}EI_{i,t-1}}{L(EI_{i,t},EI_{i,t-1}EI_{t}/EI_{t-1})}}{\sum_{i=1}^{I}\sum_{j=1}^{J}\frac{\omega_{ij}^{s-v}EI_{i,t-1}}{L(EI_{i,t},EI_{i,t-1}EI_{t}/EI_{t-1})}}$$

where $\sum_{j=1}^{J}r_{ij}(\frac{EI_{i,t}}{EI_{i,t-1}}-1)$ represents the contribution of sector *i* to the variation of energy intensity over the period [*t* − 1, *t*]; *r_ij_* denotes the weight of energy type *j* in sector *i*.

According to the single-period attribution results, the contribution of each sector to variations in energy intensity over the period [0, *T*] are expressed as:

(12) $$\frac{EI_{t}}{EI_{0}}-1=\sum_{i=1}^{I}\sum_{j=1}^{J}\sum_{t=1}^{T}\frac{EI_{t-1}}{EI_{0}}r_{ij,t-1,t}(\frac{EI_{i,t}}{EI_{i,t-1}}-1)\;r_{ij,t-1,t}=\frac{\frac{\omega_{ij,t-1,t}^{s-v}EI_{i,t-1}}{L(EI_{i,t},EI_{i,t-1}EI_{t}/EI_{t-1})}}{\sum_{i=1}^{I}\sum_{j=1}^{J}\frac{\omega_{ij,t-1,t}^{s-v}EI_{i,t-1}}{L(EI_{i,t},EI_{i,t-1}EI_{t}/EI_{t-1})}}$$

where $\sum_{j=1}^{J}\sum_{t=1}^{T}\frac{EI_{t-1}}{EI_{0}}r_{ij,t-1,t}(\frac{EI_{i,t}}{EI_{i,t-1}}-1)$ represents the contribution of sector *i* to the multi-period variations in energy intensity effect over the period [*t* − 1, *t*]. Similarly, Equations (11) and (12) can be used to describe the contribution of a sector to variations in other influencing factors.

### 2.3. Decoupling Index

Most researchers have applied the decoupling model to confirm whether environmental issues have been decoupled from economic growth [55,56,57]. At present, there are two widely used models in this field. Compared with the Organization for Economic Co-operation and Development (OECD) decoupling model, the Tapio decoupling model is more widely used due to low data requirements, simple operation, and clear results. The Tapio decoupling model is used in this study to analyze if CO_2_ emissions of these key sectors have been decoupled from industrial output.

(13) $$\gamma =\frac{\Delta C/C}{\Delta Y/Y}$$

where *γ* denotes the decoupling index between CO_2_ emissions and industrial output; *Y* represents industrial output; △*C* and △*Y* denote the variations in CO_2_ emissions and industrial output, respectively. Different levels of decoupling state are presented in Table 1.

### 2.4. Data and Study Area

The study period ranges from 2010 to 2019 considering the availability of the latest data for each provincial region. Overall, the study period can reflect the situations in the 12th and 13th five-year plan periods. Due to the absence of economic data for subdivided industrial sectors, LMDI-attribution analysis and decoupling analysis are only carried out until 2017. The data on energy consumption and CO_2_ emissions during this period are obtained from the datasets published by China Emission Accounts and Datasets (CEADs) inventory [59,60,61]. The data on industrial output and population of 30 provincial regions are gathered from the China Statistical Yearbooks [62]. To accommodate the price inflation, the industrial output data are normalized at the 2010 constant price.

The level of industrial development in China is uneven across regions, with Guangdong, Jiangsu, Shandong, Zhejiang, and other eastern coastal regions having a higher level than the central and western regions. However, the pace of industrial development in the central and western regions has been accelerated, in part supported by enhanced policies and capital investment. Meanwhile, each region has its own primary resources for development, for example, coal mining resources are concentrated in Shanxi, Inner Mongolia, Shaanxi, and other regions; Heilongjiang, Shandong, and Liaoning are richer in oil resources. In general, there are regional differences in the economy and resources; as a result, it is of great significance to analyze key sectors at the provincial level.

## 3. Results

### 3.1. Decomposition Analysis of Industrial CO_2_ Emissions

**Figure 1:**
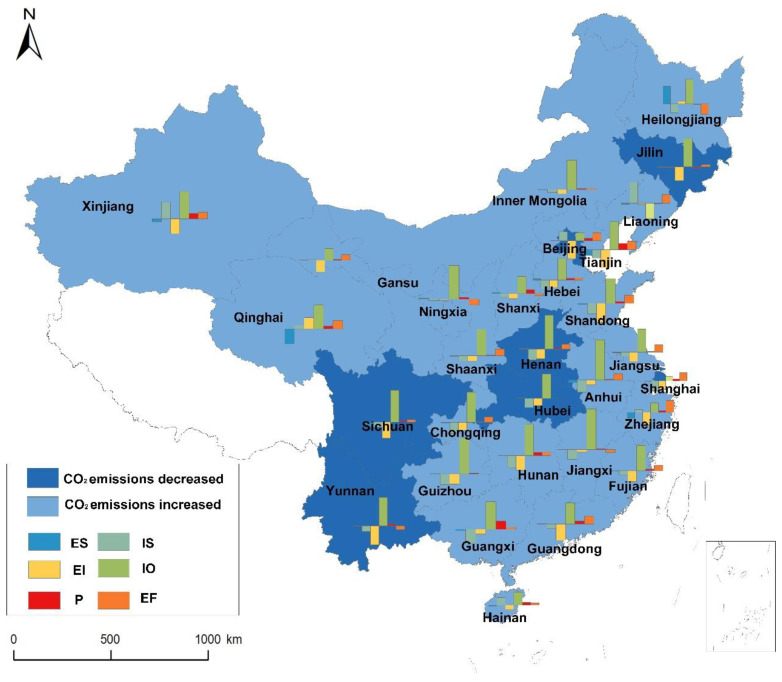
LMDI decomposition results of industrial CO_2_ emissions of regions during 2010–2017. ES: energy structure; IS: industrial structure; EI: energy intensity; IO: per capita industrial development level; P: population.

The industrial CO_2_ emissions of 30 regions are decomposed by LMDI method according to Equations (1)–(10). The results show that, in most regions, industrial development and population have positive effects, while energy intensity and industrial structure have negative effects, as illustrated in Figure 1. Industrial development is the most important driver of emissions. The regions where the contribution of industrial development is larger include Anhui, Guizhou, Jiangxi, Ningxia, Shaanxi, etc. These regions have a weaker economic base, but are relatively rich in resources to boost industrial development. However, the growth rate of industrial output of the developed regions, such as Beijing, Guangdong, Shanghai, and Zhejiang, is slowing down due to stronger economic foundation. The promotive effects of industrial development in these regions are not very obvious. The contribution of population is weaker than that of industrial development. Except for Heilongjiang, Jilin, and Liaoning, population contributes positively to emissions in most regions. In recent years, the low birth rate and serious outflow of population in the Northeast China are responsible for this situation.

Attributed to the goals for energy intensity and CO_2_ emission intensity control proposed successively in the 11th and 12th five-year plans, the effects on energy conservation and emission reduction are gradually emerging, which impels the inhibitive effect of energy intensity in most regions (except Heilongjiang and Qinghai) during the study period. Industrial structure is also an inhibitive factor in most regions. However, industrial structure in regions, such as Hainan, Liaoning, and Xinjiang, promotes emissions. Taking Liaoning as an example, the contribution rate of industrial structure is as high as 53%. This is possibly triggered by that the heavy industrial sectors with larger emissions, such as *SPFM*, have an upward trend in industrial output (the abbreviations of sectors are provided in Table 2). Meanwhile, the proportion of industrial output of the light industrial sectors is declining. As a result, industrial structure has a great promotive effect in Liaoning. As for energy structure, the inhibitive effect is slightly obvious in regions rich in renewable energy, such as Qinghai, Guangxi, and Yunnan. However, seen overall, the effect of energy structure is insignificant.

### 3.2. Attribution Analysis of Sectors’ Contributions to CO_2_ Mitigation

**Figure 2:**
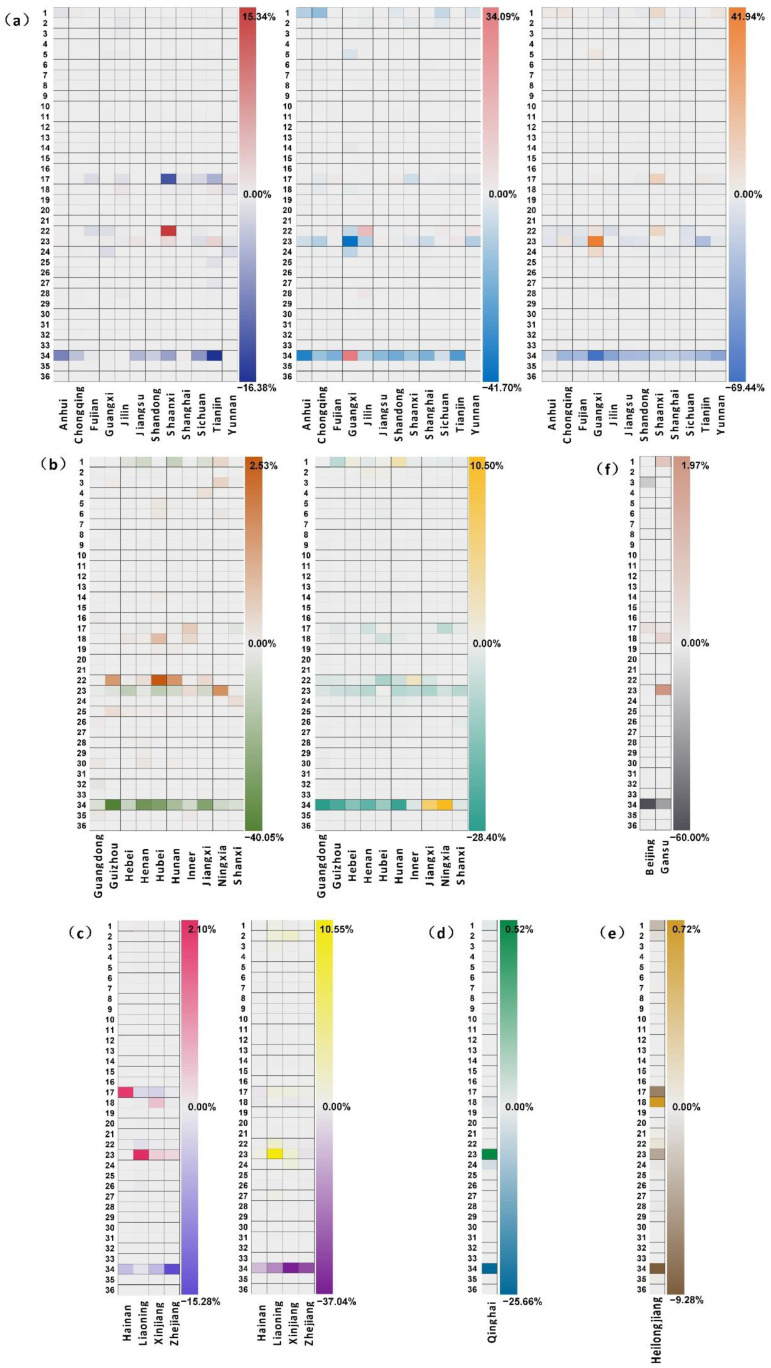
Attribution of CO_2_ mitigation to the industrial sectors through energy structure, industrial structure, and energy intensity. (**a**) Group 1 (the inhibitive factors are energy structure, industrial structure, and energy intensity). (**b**) Group 2 (the inhibitive factors are industrial structure and energy intensity). (**c**) Group 3 (the inhibitive factors are energy structure and energy intensity). (**d**) Group 4 (the inhibitive factor is energy structure). (**e**) Group 5 (the inhibitive factor is industrial structure). (**f**) Group 6 (the inhibitive factor is energy intensity).

According to the results of LMDI decomposition, energy structure, industrial structure, and reduction in energy intensity contribute significantly to CO_2_ mitigation in most regions. However, there are still some differences in specific inhibitive factors across regions. For example, the above three factors all have an inhibitive effect on CO_2_ emissions in Anhui, while only industrial structures and reduction in energy intensity contribute to CO_2_ mitigation in Guangdong. Therefore, 30 regions are divided into six groups according to the specific inhibitive factors for each region. On this basis, the variations in the effects of energy structure, industrial structure and energy intensity are further attributed to 36 subdivided industrial sectors in each region. Figure 2 combined with Tables S1–S3 in Supplementary Material shows the attribution results.

Regions in Group 1 include Anhui, Chongqing, Fujian, Guangxi, Jilin, Jiangsu, Shandong, Shaanxi, Shanghai, Sichuan, Tianjin, and Yunnan, in which energy structure, industrial structure, and energy intensity all make a contribution to emission reduction. The contribution of *PSPH* is −16.38% though energy structure in Tianjin, and that of *PPC* is −13.19% in Shaanxi, which are obviously higher than that of other sectors. Sectors that significantly contribute to emission reduction through industrial structure in Group 1 appear to *SPFM* in Guangxi (−41.7%) and *PSPH* in Anhui (−35.91%). Sectors contributing to the effects of energy intensity are basically concentrated in *PSPH* in each region, with the highest contribution in Guangxi (−69.44%). Industrial structure and energy intensity contribute to CO_2_ mitigation in regions in Group 2, including Guangdong, Guizhou, Hebei, Henan, Hubei, Hunan, Inner Mongolia, Jiangxi, Ningxia, and Shanxi. *PSPH* is the main contributor to the variations in industrial structure, followed by *CMD* in Henan (−7.49%) and Hunan (−10.15%), and *SPFM* in Hebei (−11.91%) and Hubei (−11.85%). *PSPH* in Hunan (−17.66%) makes a greater contribution to variations in energy intensity, while *SPFM* in this group promotes CO_2_ mitigation through energy intensity changes compared with Group 1. Group 3 includes Hainan, Liaoning, Xinjiang, and Zhejiang, where energy structures and energy intensity changes are the main inhibitive factors. *PSPH* is the main contributor to emission reduction in this group, whatever through which factors. Meanwhile, *PPC* in Liaoning (−1.87%) and Xinjiang (−2.62%) also have a great impact. Qinghai is the only one region in Group 4, where the contribution of *PSPH* to variations in energy structure is −25.66%. *PSPH* (−9.28%) and *PPC* (−6.80%) in Heilongjiang contribute to CO_2_ mitigation through industrial structure only, and this region is divided into Group 5. Energy intensity is the only inhibitive factor in Group 6. *PSPH* in Beijing (−60.00%) and Gansu (−28.72%) are the main contributors.

### 3.3. Key Industrial Sectors for CO_2_ Mitigation

**Figure 3:**
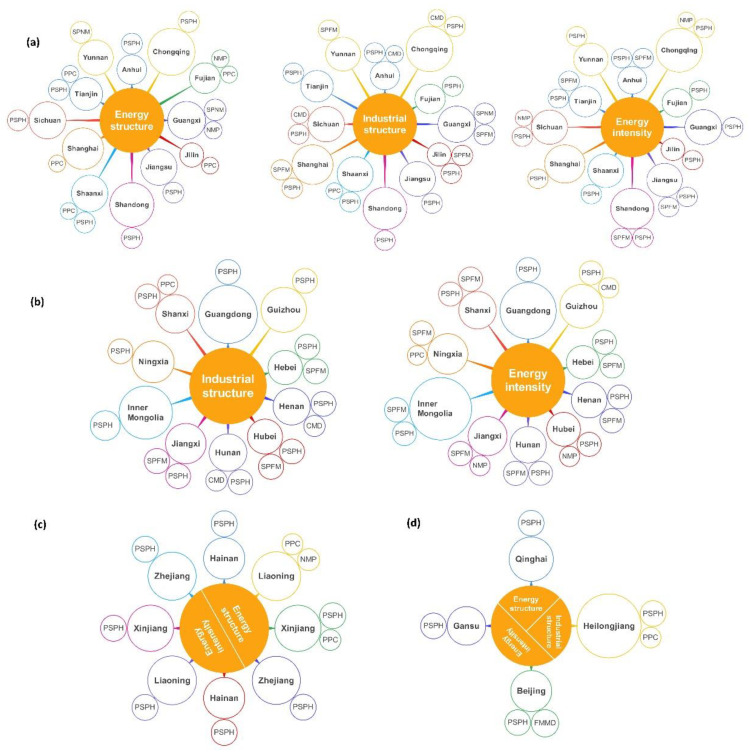
Key industrial sectors for CO_2_ mitigation in each region. (**a**) Group 1 (the inhibitive factors are energy structure, industrial structure and energy intensity). (**b**) Group 2 (the inhibitive factors are industrial structure and energy intensity). (**c**) Group 3 (the inhibitive factors are energy structure and energy intensity). (**d**) Group 4, 5, and 6 (the inhibitive factors are energy structure, industrial structure, and energy intensity, respectively). Each string of connected circles in the figure indicates that the sector (the smallest circle) has an inhibitive effect on regional (the middle circle) industrial CO_2_ emissions through an inhibitive factor (the largest orange circle).

Sectors that considerably propel CO_2_ mitigation through the inhibitive effects are defined as the key sectors in this study, which are the main forces of emission reduction deserving more attention in policy-making. However, the key sectors under different inhibitive factors are diverse, thus forming a combination of regional key sectors, as shown in Figure 3.

From the perspective of inhibitive factors, the key sectors under energy structure in Group 1 are concentrated in *PPC* and *PSPH*, while the key sectors under industrial structure and energy intensity are concentrated in *SPFM* and *PSPH*. The inhibitive factors of Group 2 are industrial structure and energy intensity, with the key sectors also concentrated in *SPFM* and *PSPH*. In Group 3, *PSPH* is the key sector for all regions under energy intensity, and the key sectors under energy structure also include *PPC* and *PSPH*. There is only one inhibitive factor in Group 3 to Group 5, with *PSPH* as the main key sector in three groups.

From the perspective of sectors, *PSPH* is the key sector for all regions, indicating that, with the development of low-carbon power supply and decarbonization technologies, CO_2_ mitigation in *PSPH* is being promoted properly. It still has a great potential for further CO_2_ mitigation that will be the main force for deepening emission reduction in energy system in the future. *PPC* and *SPFM* are also the key sectors for most regions. However, *PPC* generally contributes to emission reduction through energy structure, while *SPFM* through industrial structure and energy intensity. *SPFM* solidifies energy intensity improvement through energy-saving technological transformation, such as reducing the use of coal for iron making and smelting, and expanding clean energy use. *PPC* accelerates energy structure adjustment through eliminating outdated production capacity, combined with technological advancement, such as augmenting biofuel production. Other sectors, such as *CMD* in Anhui, Sichuan, Chongqing, Henan, and Hunan promote emission reduction mainly through industrial structure adjustment. *NMP* contributes to CO_2_ mitigation mainly in Fujian, Sichuan, Chongqing, and Hubei through energy intensity changes. *SPNM* is the main promoter of emission reduction in Guangxi and Yunnan.

### 3.4. Emission Characteristics of the Key Sectors for CO_2_ Mitigation

The critical emission characteristics, including decoupling from industrial development and the peaking status in each region are analyzed so as to better compare the emission status of the key sectors in different regions, with the results presented in Table 3. The two columns under each sector represent the two emission characteristics. Seen overall, in terms of either the number of key sectors that contribute to CO_2_ mitigation or the emission characteristics, the performance of the economically developed eastern coastal regions is overall better than that of the economically underdeveloped central and western regions.

From the perspective of sector, except *CMD* whose emissions present recessive decoupling or negative decoupling, other key sectors in most regions have accomplished the decoupling of emissions from industrial development. However, through the comparison of the emission peaking status of regions, *CMD* is the one with the best situation among the key sectors, while *SPFM* and *PSPH* in most regions have not peaked the emissions, especially *PSPH*, whose emissions in most regions are still on the rise. From the perspective of region, Beijing and Shanghai have a small energy intensity although they have maintained the rapid growth in industrial output, and the decoupling from industrial development and the peaking status of the key sectors have been basically achieved through industrial restructure. Emissions of most key sectors in Fujian, Jiangsu, Sichuan, and Tianjin have been decoupled from industrial development, however without showing a clear trend towards peaking. Hebei, Hubei, Jilin, and Shaanxi are heavy industrial regions in need for transformation. The emissions of the key sectors have already turned a corner, but present weak negative decoupling due to the downward growth rate of industrial output. Some western regions, such as Guangxi and Xinjiang, fall behind in industrial development. Emissions of the key sectors are in a state of negative decoupling, and the emission peaking is yet to be realized.

## 4. Discussion

The attribution analysis reveals that sectors contributing to CO_2_ mitigation across regions are mainly concentrated in *PSPH, SPFM,* and *PPC,* which is attributed to reinforced government’s control over these sectors in recent years. Alongside energy efficiency improvement and renewable energy development, these key sectors have basically realized the decoupling from industrial development. While promoting CO_2_ mitigation, *PSPH* also needs to meet the continuous growth of power demand. However, restricted by the coal-dominated energy structure, the emissions of *PSPH* lag far from the peak, which is in compliance with the results of Wen et al. [63]. The same situation happens to *SPFM*, which is the second largest emitter following *PSPH* due to its large scale and production process characteristics relying on fossil fuels.

There are differences in the key sectors and emission characteristics among regions. Beijing, Qinghai, Shanghai, and Sichuan are among the minority of regions where *PSPH* has achieved the emission peak. These regions are either at the forefront of economic development or rich in clean energy. The promotion of power trading markets, and the deployment of pumped storage and offshore power stations have prompted to improve power generation efficiency. *CMD* is the key sector for Anhui, Chongqing, Henan, Hunan, and other regions, in addition to the above sectors. The number of mines with small single wells in these regions have reduced since the implementation of coal supply-side reform because of the poor mining conditions. Meanwhile, the support of relevant policies, such as controlling the total number of coal mines in Hunan, resolving overcapacity in Henan, as well as putting forward coal-intelligence goals in Anhui and Henan, have also contributed to CO_2_ mitigation in this sector. However, the decoupling status of *CMD* is mainly in recessive coupling, indicating that both CO_2_ emission and industrial output are declining, which shows the main task of the sector in these regions is changing the development mode to realize the decoupling as soon as possible. *PPC* mainly contribute to CO_2_ mitigation in the Northeast China and some oil-producing regions, such as Shaanxi, Tianjin, and Xinjiang, among which the decoupling has been achieved, except Jilin and Shaanxi. The long industrial chain, the wide variety of products, and the continuous growth of oil consumption pose severe challenges to low-carbon transition in this sector. Strengthening emission reduction in production process and alleviating energy consumption caused by raw materials to achieve deeper decarbonization is the key to reaching the peak for the sector. There are unique non-metallic resources in Hunan, Jiangxi, and Sichuan, and the industrial parks have been established successively in these regions. The development of energy efficient materials, implementation of access standards and establishment of R&D centers have facilitated a green manufacturing system, enabling *NMP* to promote CO_2_ mitigation in these regions. *SPNM* undergoes high-quality development in regions rich in mineral resources, such as Yunnan and Guangxi, through technological breakthrough as in the case of aluminum electrolysis.

The methods presented in this paper that couple the inhibitive factors extracted from LMDI analysis with attribution analysis provide an approach capable of quantifying the contribution of industrial sectors to the drivers and extracting the key sectors that contributed to CO_2_ mitigation. Complex network approach and input–output model have also been applied to analyze the different roles of various sectors to help decision-makers identify key sectors [64,65]. However, the key sectors identified in this study are those under the inhibitive factors that promote CO_2_ mitigation in each region, meaning that these sectors are already contributing to emission reductions. Additionally, this is a better visualization of which factors of these sectors can be adjusted to mitigate regional CO_2_ more effectively, and facilitate policymakers to develop more targeted emission reduction policies. Furthermore, this study analyzes the critical emission characteristics of the key sectors across regions, so as to provide reference to formulating differentiated emission reduction measures for regions with different levels of development, policies, and technological guidance. The methods can be extended to recognize the key sectors in terms of significant contribution to the emission and mitigation of air pollutants or water pollutants.

Some policy implications can be revealed through combing the strategic goal of China’s carbon peaking before 2030 with the findings of this study. Firstly, compared with other sectors, *PSPH* and *SPFM* in most regions have not yet achieved the peak. Renewable energy should be the priority for power generation in the future, meanwhile the energy storage technology and power market should be extended to promote an early emission peak for *PSPH.* As for *SPFM*, excessive and outdated production capacity is still the main problem faced by this sector. Secondly, while *PPC* is undergoing prosperous development in Tianjin, Heilongjiang, Xinjiang, and other oil-producing regions, it still suffers from heavy emission control burden. In order to arrive at the emission peak earlier, adjusting the structure of refinery products and replacing with greener fuels is the prior mission. *CMD* has made certain contribution to CO_2_ mitigation in Anhui, Hunan, Henan, and other regions. However, this sector should be further focused on in Shanxi, Shaanxi, and Mongolia for the release of production capacity of mines under construction and the elimination of mines with poor endowments. Regions rich in mineral resources, such as Yunnan and Guangxi, should strengthen technological innovation and policy guidance in *SPNM.* Finally, regions, such as Beijing, Guangdong, and Zhejiang, only have *PSPH* as their key sector. As the first echelon leading China in achieving the carbon peaking and carbon neutrality goals, they should keep transforming their own advantages into the tractive force for the development of the surrounding regions and provide support to boost low-carbon development in surrounding regions.

## 5. Conclusions

In the context of pursuing earlier carbon peaking in China, CO_2_ mitigation in industry, which the largest source of CO_2_ emissions, is a foremost task. Using the LMDI-attribution method, this study identifies the key sectors that significantly contribute to CO_2_ mitigation in 30 provincial regions in China, and analyzes the emission characteristics, including decoupling from industrial development and peaking status of these sectors. Several key findings are summarized as follows:

Industrial development and population growth are the main drivers of emissions. Energy intensity and industrial structure inhibit emissions in most regions, but industrial structure is a main factor promoting emissions in some regions as result of the increasing share of energy-intensive sector. The effect of energy structures on emission reduction in most regions is not obvious. According to the combinations of inhibitive factors, the 30 regions are divided into six groups. The key sectors that promote CO_2_ mitigation in each region are tracked through the inhibitive effects, mainly including: *coal mining and dressing*, *petroleum processing and coking, non-metal mineral products*, *smelting and pressing of ferrous metals, smelting and pressing of nonferrous metals,* and *production and supply of power and heat.* Of these, *production and supply of power and heat* propels CO_2_ mitigation through all three inhibitive factors. *Petroleum processing and coking* generally contributes to emission reduction through energy structure, while *smelting and pressing of ferrous metals* through industrial structure and energy intensity.

The results of the study also show the emission characteristics of key sectors in each region. *Production and supply of power and heat* is the key sector in all regions, followed by *smelting and pressing of ferrous metals*, but these two sectors in most regions have not achieved the emission peak. Except *coal mining and dressing*, the key sectors in most regions have accomplished the decoupling of emissions from industrial development. In addition, *coal mining and dressing* effectively promotes CO_2_ mitigation in Anhui, Henan, and Hunan, with a larger contribution of *petroleum processing and coking* in Tianjin, Xinjiang, Heilongjiang, and other oil-producing regions, and that of *smelting and pressing of nonferrous Metals* in Yunnan and Guangxi.

This study identifies the key sectors that contribute to regional emission reductions, which are major areas for future industrial CO_2_ mitigation; it also examines the critical emission characteristics of these sectors. The level of development of the key sectors varies from region to region, and the results of the study can provide assistance in formulating regional emission reduction paths that are more suitable for local development. The revealed policy implications can serve for better policy-making on sector-level and region-level carbon mitigation practices. There are still some limitations in this paper. First of all, due to the limitations of the research method of attribution analysis, the change of the total amount can only be decomposed into one-dimensional factors, which makes it impossible to explore the driving factors of the change of emissions from multiple dimensions, such as different sectors and different energy types. Second, the key sectors discussed in this study are those under the inhibitive factors of industrial CO_2_ emissions, and the promotive factors of emissions are not specific to the segmented sectors, which should be incorporated into the further work.

## Figures and Tables

**Table 1:** Levels of decoupling state [58].

△*C*/*C*	△*Y*/*Y*	*γ*	Decoupling State
<0	>0	*γ* < 0	Strong decoupling
>0	>0	0.8 > *γ* ≥ 0	Weak decoupling
>0	>0	1.2 ≥ *γ* ≥ 0.8	Expansive coupling
>0	>0	*γ* > 1.2	Expansive negative decoupling
>0	<0	*γ* < 0	Strong negative decoupling
<0	<0	0.8 > *γ* ≥ 0	Weak negative decoupling
<0	<0	1.2 ≥ *γ* ≥ 0.8	Recessive coupling
<0	<0	*γ* > 1.2	Recessive decoupling

Strong decoupling: CO_2_ emissions decrease while GDP increases; Weak decoupling: CO_2_ emissions are growing more slowly than GDP; Expansive decoupling: CO_2_ emissions increase in step with GDP; Expansive negative decoupling: CO_2_ emissions are growing much faster than GDP; Strong negative decoupling: CO_2_ emissions increase while GDP decreases; Weak negative decoupling: CO_2_ reduction is slower than GDP recession; Recessive coupling: CO_2_ emissions are declining at the same speed as GDP; Recessive decoupling: CO_2_ emissions are falling much faster than GDP.

**Table 2:** Sector classification and abbreviation.

Code	Sector	Abbreviation
**1**	***Coal Mining and Dressing***	***CMD***
2	Petroleum and Natural Gas Extraction	PNGE
3	Ferrous Metals Mining and Dressing	FMMD
4	Nonmetal Minerals Mining and Dressing	NMMD
5	Food Processing	FPS
6	Food Production	FP
7	Beverage Production	BP
8	Tobacco Processing	TP
9	Textile Industry	TI
10	Garments and Other Fiber Products	GOFP
11	Leather, Furs, Down and Related Product	LFDRP
12	Timber Processing, Bamboo, Cane, Palm Fiber, and Straw Products	TPBCP
13	Furniture Manufacturing	FM
14	Papermaking and Paper Products	PPP
15	Printing and Record Medium Reproduction	PRMR
16	Cultural, Educational and Sports Articles	CESA
**17**	***Petroleum Processing and Coking***	***PPC***
18	Raw Chemical Materials and Chemical Products	RCMCP
19	Medical and Pharmaceutical Products	MPP
20	Chemical Fiber	CF
21	Rubber and Plastic Products	RPP
**22**	***Nonmetal Mineral Products***	***NMP***
**23**	***Smelting and Pressing of Ferrous Metals***	***SPFM***
**24**	***Smelting and Pressing of Nonferrous Metals***	***SPNM***
25	Metal Products	MP
26	Ordinary Machinery	OM
27	Equipment for Special Purposes	ESP
28	Transportation Equipment	TE
29	Electric Equipment and Machinery	EEM
30	Electronic and Telecommunications Equipment	ETE
31	Instruments, Meters, Cultural and Office Machinery	IMCOM
32	Other Manufacturing Industry	OMI
33	Scrap and waste	SW
**34**	***Production and Supply of Power and Heat***	***PSPH***
35	Production and Supply of Gas	PSG
36	Production and Supply of Tap Water	PSTW

**Table 3:** Critical emission characteristics of the key industrial sectors of regions.

		Coal Mining and Dressing		Petroleum Processing and Coking		Nonmetal Mineral Products		Smelting and Pressing of Ferrous Metals		Smelting and Pressing of Nonferrous Metals		Production and Supply of Power and Heat	
**Group 1**	**Anhui**	RC	○					WD	x			WD	x
	**Chongqing**	WND	○			WD	○					WD	x
	**Fujian**			WD	x	SD	○					SD	x
	**Guangxi**							WD	x	SND	x	SD	x
	**Jilin**			EC	○			SD	x			SND	x
	**Jiangsu**							WD	x			WD	x
	**Shandong**							SD	x			WD	x
	**Shaanxi**			END	○							WD	x
	**Shanghai**			SD	x			RD	○			SD	○
	**Sichuan**	RD	○			SD	x					SD	○
	**Tianjin**			SD	x			SD	x			SD	x
	**Yunnan**							RD	x	SD	x	SD	x
**Group 2**	**Guangdong**											SD	x
	**Guizhou**	SD	○									WD	x
	**Hebei**							WD	x			WND	x
	**Henan**	RC	○					WD	○			SD	x
	**Hubei**					SD	○	WND	x			SD	x
	**Hunan**	RC	○					SD	x			SD	x
	**Jiangxi**					WD	○	WD	x			WD	x
	**Inner Mongolia**							SD	x			END	x
	**Ningxia**			SD	x			WD	x			WD	x
	**Shanxi**			SD	○			SD	x			WD	x
**Group 3**	**Hainan**											WD	x
	**Liaoning**			RD	x	RC	x					WD	x
	**Xinjiang**			RD	x							EC	x
	**Zhejiang**											WD	x
**Group 4**	**Beijing**											SD	○
	**Gansu**											SD	x
**Group 5**	**Heilongjiang**			RD	○							WD	x
**Group 6**	**Qinghai**											WD	○

SD (strong decoupling); WD (weak decoupling); EC (expansive coupling); END (expansive negative decoupling); SND (strong negative decoupling); WND (weak negative decoupling); RC (recessive coupling); RD (recessive decoupling); ○: with an emission peak; x: without an emission peak.

## Supplementary Materials

The following supporting information can be downloaded at: https://www.mdpi.com/article/10.3390/ijerph192114561/s1, Table S1. Multi-period attribution analysis of energy structure; Table S2. Multi-period attribution analysis of industrial structure; Table S3. Multi-period attribution analysis of energy intensity.
