# Supplementary material for: Tracking Key Industrial Sectors for CO2 Mitigation through the Driving Effects: An Attribution Analysis
Source: Int J Environ Res Public Health. 2022 Nov 7;19(21):14561. doi: 10.3390/ijerph192114561 (PMC9653607; doi:10.3390/ijerph192114561)
Supplement: Supplementary file 1 [file ijerph-19-14561-s001.zip › ijerph-1918686-supplementary.pdf]

Supplementary material for

Tracking key industrial sectors for CO_2 mitigation through the driving effects: An attribution analysis

Xian'en Wang [1,2,3], Tingyu Hu [1,2], Junnian Song [1,2,3,*], Haiyan Duan [1,2,3]

[1] Key Laboratory of Groundwater Resources and Environment, Ministry of Education, Jilin University, Changchun 130021, China.

[2] College of New Energy and Environment, Jilin University, Changchun 130012, China.

[3] Jilin Provincial Key Laboratory of Water Resources and Environment, Jilin University, Changchun 130021, China.

***Corresponding author:**

Junnian Song, songjunnian@jlu.edu.cn, 2699 Qianjin Street, Jilin University, 130012 Changchun, China.

Table S1. Multi-period attribution analysis of energy structure.

Table S2. Multi-period attribution analysis of industrial structure.

Table S3. Multi-period attribution analysis of energy intensity.

Table S1. Multi-period attribution analysis of energy structure (Unit: %).

Sector	Anhui	Beijing	Chongqing	Fujian	Gansu	Guangdong	Guangxi	Guizhou	Hainan	Hebei	Henan	Heilongjiang	Hubei	Hunan	Inner Mongolia
CMD	-0.95	0.00	0.98	-0.02	-0.09	0.00	0.00	0.13	0.00	1.06	1.05	-0.22	0.00	0.05	0.05
PNGE	0.00	0.07	0.00	0.00	-0.07	0.00	0.00	0.00	0.00	0.01	-0.12	0.15	0.00	0.00	0.00
FMMD	-0.02	-0.82	0.00	-0.03	-0.07	0.02	-0.02	0.00	0.00	-0.01	0.04	0.00	-0.02	0.01	0.06
NMMD	-0.04	0.00	-0.07	-0.05	0.04	0.01	-0.01	0.00	0.00	0.00	0.01	-0.03	-0.01	0.03	0.01
FPS	-0.07	0.05	-0.02	0.01	0.07	0.00	-0.29	0.00	0.01	0.01	-0.03	0.52	-0.29	-0.02	0.01
FP	-0.04	0.02	0.02	-0.07	0.00	-0.02	-0.01	0.00	-0.05	0.00	-0.03	0.09	-0.01	-0.06	0.01
BP	-0.10	-0.09	-0.03	-0.01	-0.04	0.00	-0.04	-0.01	0.01	0.00	-0.04	0.09	-0.04	0.00	0.00
TP	0.00	0.00	0.00	0.00	0.00	0.00	0.00	0.00	0.00	0.00	0.00	-0.01	0.00	0.15	0.00
TI	-0.01	0.00	-0.03	-0.09	0.00	0.07	-0.04	0.00	0.00	0.00	-0.07	0.00	-0.04	0.00	0.00
GOFP	-0.01	0.01	0.00	0.00	0.00	0.00	0.00	0.00	0.00	0.00	0.00	0.00	0.00	0.00	0.00
LFDRP	0.00	0.00	0.00	0.01	0.00	0.00	0.00	0.00	0.00	0.00	-0.03	0.00	0.00	-0.02	0.00
TPBCP	0.00	0.00	0.00	-0.02	0.00	0.00	-0.01	0.00	0.00	0.00	-0.02	0.01	-0.01	-0.04	0.00
FM	0.00	-0.04	0.00	0.00	0.00	-0.01	0.00	0.00	0.00	0.00	0.00	-0.01	0.00	0.00	0.00
PPP	-0.02	0.01	-0.03	-0.07	-0.01	0.09	-0.02	0.02	-0.08	0.00	0.00	0.00	-0.02	-0.03	0.00
PRMR	-0.01	0.01	0.00	0.00	0.00	-0.01	0.00	0.00	0.00	0.00	0.00	0.00	0.00	-0.01	0.00
CESA	0.00	0.01	0.00	0.01	0.00	-0.10	0.00	0.00	0.00	0.00	0.00	0.00	0.00	0.00	0.00
PPC	0.07	1.30	-0.12	-1.56	0.92	0.08	-0.05	0.20	1.96	0.43	0.97	-3.44	-0.05	-0.69	0.12
RCMCP	-0.01	-0.74	-0.07	0.06	0.29	-0.21	-0.04	0.39	0.03	-0.08	0.00	5.17	-0.04	-0.09	-0.53
MPP	0.00	0.02	-0.08	0.01	0.00	-0.01	0.00	0.00	0.00	0.00	0.02	-0.10	0.00	-0.04	0.00
CF	0.01	0.00	0.00	-0.01	0.00	-0.01	0.00	0.00	0.00	0.00	-0.02	0.00	0.00	0.00	0.00
RPP	0.16	-0.01	-0.01	0.02	0.00	-0.04	-0.02	0.00	-0.10	0.00	-0.02	0.00	-0.02	0.03	0.07
NMP	-0.04	-0.40	-0.01	-1.51	-0.07	0.24	-1.28	-0.27	0.03	-0.06	-0.30	0.09	-1.28	0.36	2.89
SPFM	0.57	-0.22	-0.04	0.59	-0.56	3.63	0.85	-0.08	0.00	1.00	2.96	2.26	0.85	1.07	-3.40
SPNM	-0.01	0.00	-0.26	0.34	0.03	0.12	-1.49	-0.06	0.00	0.00	-0.37	0.99	-1.49	-0.07	0.12

MP	-0.03	0.02	0.03	0.02	-0.02	-0.04	0.01	0.01	0.00	-0.01	-0.01	-0.03	0.01	0.03	-0.01
OM	-0.29	0.01	-0.02	0.06	0.00	0.04	0.02	0.00	0.00	-0.01	-0.10	-0.01	0.02	-0.05	0.00
ESP	-0.02	0.02	0.01	-0.02	0.00	0.00	0.00	0.00	0.00	0.00	-0.06	0.37	0.00	-0.12	0.00
TE	-0.13	0.15	-0.14	0.00	0.00	-0.02	-0.03	0.00	0.01	-0.01	-0.14	-0.21	-0.03	-0.04	0.00
EEM	-0.08	0.01	0.00	0.00	0.00	-0.03	0.00	0.00	0.00	0.00	0.00	0.00	0.00	-0.02	0.00
ETE	0.00	0.00	0.01	-0.01	0.00	-0.01	0.00	0.00	0.00	0.00	0.00	0.00	0.00	-0.09	0.00
IMCOM	0.00	0.00	0.00	0.00	0.00	-0.01	0.00	0.00	0.00	0.00	0.00	0.00	0.00	-0.04	0.00
OMI	0.00	0.02	0.00	0.13	0.00	0.11	0.00	0.00	0.00	0.00	-0.01	0.00	0.00	0.06	0.00
SW	0.01	0.00	0.00	0.00	0.06	0.00	0.00	0.00	0.00	0.00	0.00	0.00	0.00	0.00	0.00
PSPH	-9.27	2.31	-3.75	-0.45	0.08	-2.98	-0.15	-0.25	-4.26	1.26	-0.85	44.11	-0.15	-0.05	0.35
PSG	0.00	0.08	0.00	-0.02	0.00	0.00	-0.03	0.17	0.00	0.00	0.15	0.18	-0.03	0.00	0.07
PSTW	0.00	0.00	0.00	0.00	0.00	0.00	0.00	0.00	0.00	0.00	0.00	0.00	0.00	0.00	0.00

Table S1 (continued)

Sector	Jilin	Jiangsu	Jiangxi	Liaoning	Ningxia	Qinghai	Shandong	Shanxi	Shaanxi	Shanghai	Sichuan	Tianjin	Xinjiang	Yunnan	Zhejiang
CMD	-0.18	-0.02	0.02	0.04	0.21	-1.14	0.05	0.07	0.14	0.00	-0.03	0.00	-0.05	0.29	0.00
PNGE	-0.52	-0.01	0.00	0.02	0.00	-0.20	-0.02	0.00	-0.20	0.00	0.26	-0.16	-0.18	0.00	0.00
FMMD	-0.37	0.00	0.02	0.02	0.00	0.00	0.03	-0.03	0.00	0.00	-0.06	0.07	0.01	0.17	0.00
NMMD	0.02	0.00	0.11	0.03	0.00	-0.01	0.00	0.00	0.00	0.00	-0.06	0.00	0.00	-0.01	-0.01
FPS	-0.26	0.00	0.03	-0.08	0.00	-0.02	-0.01	0.00	-0.08	-0.01	-0.09	-0.15	-0.06	0.03	0.00
FP	0.00	0.00	-0.10	0.02	-0.12	0.05	0.01	-0.01	-0.15	0.00	-0.06	-0.03	-0.01	0.04	0.00
BP	-0.02	0.00	0.01	-0.01	0.00	-0.07	-0.01	0.00	-0.03	0.00	-0.10	0.01	0.00	0.01	0.00
TP	0.00	0.00	0.00	0.00	0.00	0.00	0.00	0.00	0.00	0.00	0.00	0.00	0.00	0.01	0.00
TI	0.01	-0.01	0.04	0.01	0.00	0.00	-0.02	0.00	0.00	-0.01	-0.09	0.01	-0.01	0.00	-0.01
GOFP	0.00	-0.01	0.01	0.02	0.00	0.01	-0.01	0.00	0.00	-0.02	0.00	0.01	0.00	0.00	-0.01
LFDRP	0.00	0.00	0.00	0.00	0.00	0.00	0.00	0.00	0.00	-0.01	-0.02	0.00	0.00	0.00	0.00

TPBCP	0.05	0.00	0.00	0.01	0.00	0.00	0.01	0.00	0.00	0.00	-0.02	0.00	0.00	0.00	0.00
FM	-0.02	0.00	0.00	0.01	0.00	0.00	0.00	0.00	0.00	0.00	-0.01	0.01	0.00	0.00	-0.01
PPP	-0.03	-0.01	0.06	-0.01	0.00	0.00	-0.01	0.00	0.00	0.02	-0.03	-0.04	-0.01	0.00	0.00
PRMR	0.02	0.00	0.00	0.00	0.00	0.04	0.00	0.00	0.00	-0.04	-0.04	0.14	0.00	0.00	0.00
CESA	0.00	0.00	0.00	0.00	0.00	0.00	0.00	0.00	0.00	0.00	0.00	-0.01	0.00	0.00	0.00
PPC	-1.17	0.04	0.50	-1.87	1.28	-0.10	0.12	1.69	-13.19	-0.69	-1.87	-5.19	-2.62	1.48	-0.50
RCMCP	1.72	0.21	-0.19	-0.31	-0.05	-1.32	0.16	-0.25	-0.29	-0.43	0.23	1.50	0.49	-1.08	-0.39
MPP	-0.25	0.00	0.00	-0.01	0.00	0.02	0.02	0.00	0.00	0.00	0.02	-0.02	0.00	0.00	0.00
CF	0.00	-0.01	0.00	0.02	0.00	0.00	0.01	0.00	0.00	0.00	0.00	0.00	0.00	0.00	-0.01
RPP	-0.03	0.02	0.01	0.03	0.02	0.00	0.01	0.00	0.00	-0.02	-0.04	0.17	0.00	0.00	-0.02
NMP	-0.19	-0.04	-0.24	-1.28	-0.42	-0.16	-0.19	0.00	15.34	-0.02	-0.39	0.00	-0.25	0.00	-0.23
SPFM	-0.46	1.93	0.19	2.10	0.18	0.52	0.59	-0.39	3.24	-0.14	-1.38	4.33	0.34	-0.77	0.24
SPNM	-0.06	-0.01	-0.01	-0.04	-0.08	-2.96	-0.08	0.01	0.45	0.04	-0.22	0.12	-0.08	-1.41	-0.06
MP	-0.07	0.00	0.00	-0.11	0.01	0.00	-0.01	-0.02	0.00	0.00	-0.02	-1.05	0.01	0.00	-0.03
OM	0.07	0.03	0.02	-0.48	0.00	0.04	0.00	-0.04	-0.20	-0.01	-0.07	-0.03	0.00	0.00	-0.04
ESP	-0.06	-0.01	0.00	-0.04	0.00	0.00	-0.01	0.00	-0.03	-0.02	-0.01	-0.70	0.00	0.00	-0.01
TE	-0.41	0.00	0.07	-0.05	0.00	0.00	-0.03	0.01	0.36	0.01	-0.06	-0.15	0.00	0.03	-0.05
EEM	0.01	0.00	0.02	-0.10	0.00	0.00	-0.02	0.00	0.04	0.03	-0.01	-0.05	0.00	0.00	-0.02
ETE	0.01	-0.01	0.00	0.02	0.00	0.00	0.00	-0.01	0.02	-0.01	-0.01	0.00	0.00	0.00	-0.02
IMCOM	0.00	0.00	0.00	0.00	0.00	0.00	0.00	0.00	0.00	0.00	0.00	0.00	0.00	0.00	-0.01
OMI	0.00	0.00	0.00	0.00	0.00	0.00	0.00	0.00	0.00	0.00	-0.01	0.00	0.00	0.00	-0.01
SW	0.00	0.00	0.00	0.00	0.00	0.00	0.00	0.00	0.00	0.00	0.00	0.01	0.00	-0.04	0.00
PSPH	0.00	-4.78	0.05	-0.98	3.73	-25.66	-2.86	2.17	-6.51	0.66	-7.62	-16.38	-4.70	0.13	-15.28
PSG	0.16	0.02	0.00	0.02	0.00	0.00	0.04	0.00	0.00	0.00	-0.02	0.00	-0.05	0.14	0.00
PSTW	0.00	0.00	0.00	0.00	0.00	0.07	0.00	0.00	0.00	0.00	0.00	0.00	0.00	0.00	0.00

Table S2. Multi-period attribution analysis of industrial structure (Unit: %).

Sector	Anhui	Beijing	Chongqing	Fujian	Gansu	Guangdong	Guangxi	Guizhou	Hainan	Hebei	Henan	Heilongjiang	Hubei	Hunan	Inner Mongolia
CMD	-9.81	0.00	-16.11	-0.33	-0.08	0.00	0.00	-0.10	0.00	-3.93	-7.49	-3.32	-0.32	-10.15	0.00
PNGE	0.00	0.00	-0.06	0.00	-0.02	-0.06	0.00	0.00	0.00	-0.17	-0.67	-1.01	-0.49	0.00	0.00
FMMD	0.01	-0.42	0.00	-0.10	-0.28	-0.05	-0.10	0.07	0.05	-0.06	-0.01	0.00	-0.05	-0.04	-0.01
NMMD	0.00	0.00	-0.16	0.01	-0.01	-0.02	-0.22	0.00	0.00	0.00	0.00	0.00	0.03	-0.10	-0.01
FPS	0.00	-0.02	-0.01	-0.04	-0.06	0.00	-3.49	0.00	0.00	0.02	0.00	0.21	0.14	0.04	-0.03
FP	0.01	0.01	-0.02	0.05	-0.01	0.01	0.03	0.00	-0.02	0.04	0.01	-0.06	0.17	0.03	-0.05
BP	0.00	-0.02	-0.03	0.01	-0.03	0.01	-0.20	0.04	0.00	0.01	0.00	0.02	0.04	0.02	0.00
TP	0.00	0.00	0.00	0.00	0.00	0.00	0.00	-0.03	0.00	0.00	0.00	0.00	0.00	-0.02	0.00
TI	0.00	0.00	-0.08	0.13	0.00	-0.53	-0.16	0.00	0.00	0.01	-0.01	0.06	0.03	-0.04	-0.01
GOFP	0.00	0.00	0.00	-0.03	0.00	0.01	0.00	0.00	0.00	0.00	0.00	0.00	0.01	0.00	0.00
LFDRP	0.00	0.00	0.00	0.00	0.00	-0.01	-0.01	0.00	0.00	0.01	0.00	0.00	0.00	0.02	0.00
TPBCP	0.00	0.00	0.00	0.00	0.00	0.00	-0.20	0.00	0.00	0.01	0.00	0.05	0.01	-0.03	0.00
FM	0.00	-0.01	0.00	0.00	0.00	0.00	0.00	0.00	0.00	0.01	0.00	0.00	0.00	0.00	0.00
PPP	-0.03	0.00	-0.19	0.01	0.01	-0.07	-1.52	-0.01	0.66	-0.01	-0.12	-0.10	0.09	-0.23	0.00
PRMR	0.00	0.00	0.00	0.01	0.00	-0.02	0.00	0.00	0.00	0.00	0.00	0.00	0.00	0.00	-0.02
CESA	0.00	0.00	0.00	0.01	0.00	0.09	0.00	0.00	0.00	0.00	0.00	0.02	0.00	0.00	0.00
PPC	-0.10	-5.41	-1.90	1.81	-1.30	-0.57	-0.10	-0.67	0.51	-0.45	-2.12	-6.80	-0.32	-1.30	0.59
RCMCP	-0.01	-0.17	-1.24	0.02	-0.56	-0.05	-0.94	-0.16	1.10	0.17	0.07	0.72	0.83	-0.30	0.36
MPP	0.00	0.02	0.01	0.00	0.00	0.00	0.01	0.00	0.01	0.00	0.01	-0.02	0.09	0.09	0.00
CF	0.00	0.00	0.00	0.01	0.09	-0.01	0.00	0.00	0.00	0.00	-0.03	-0.08	0.00	0.00	0.00
RPP	0.00	0.00	0.00	-0.04	0.00	-0.05	0.01	0.00	0.00	0.01	0.00	0.03	0.00	0.00	0.00
NMP	0.62	-0.50	-0.25	1.07	0.73	-0.10	-7.27	1.44	0.68	0.05	0.28	0.08	2.53	1.53	-0.31
SPFM	-5.12	-0.86	-9.97	-0.51	-6.54	-0.97	-41.70	-3.85	-0.66	-11.91	-2.72	-4.53	-11.85	-7.16	0.31
SPNM	-0.04	0.00	-0.36	0.60	1.51	-0.10	-8.22	-0.12	0.00	0.00	-0.36	-0.33	-0.11	-0.34	0.00

MP	0.00	0.01	0.03	0.02	0.02	-0.05	0.03	0.30	-0.06	0.09	0.05	0.01	0.06	0.10	0.00
OM	0.01	0.01	-0.07	-0.01	0.00	0.09	0.01	0.00	0.00	-0.13	-0.05	-0.11	-0.13	-0.01	0.00
ESP	0.00	0.01	0.00	-0.01	-0.01	0.02	0.00	0.00	0.00	0.03	0.00	-0.25	0.02	0.01	-0.03
TE	0.00	0.35	-0.10	-0.05	0.00	0.02	0.08	0.01	0.00	0.04	0.08	-0.22	-0.15	0.00	0.00
EEM	0.00	-0.01	0.00	-0.01	0.00	-0.06	0.00	0.00	0.00	0.00	0.07	-0.01	0.01	-0.01	0.00
ETE	0.00	0.01	0.00	-0.01	0.00	0.14	0.00	0.00	0.00	0.00	0.17	0.00	0.00	0.06	0.00
IMCOM	0.00	0.00	0.00	0.00	0.00	-0.05	0.00	0.00	0.00	0.00	0.00	0.00	0.00	0.00	0.00
OMI	0.00	-0.01	0.00	-0.27	0.00	-2.79	0.00	0.00	0.00	0.00	0.00	0.11	-0.01	0.00	0.00
SW	0.00	0.00	0.00	0.00	0.01	-0.01	0.00	0.00	0.00	0.00	0.00	0.00	0.01	0.00	0.00
PSPH	-35.91	46.67	-14.26	-20.14	9.46	-4.90	34.09	-40.05	17.11	-9.83	-32.64	-9.28	-28.50	-17.66	-7.21
PSG	0.00	0.27	0.01	0.07	0.00	0.14	0.48	-0.63	0.00	0.00	0.02	0.03	0.00	0.00	0.11
PSTW	0.00	0.01	0.00	0.00	0.00	0.00	0.00	0.00	0.00	0.00	0.00	-0.02	0.00	0.00	0.00

Table S2 (continued)

Sector	Jilin	Jiangsu	Jiangxi	Liaoning	Ningxia	Qinghai	Shandong	Shanxi	Shaanxi	Shanghai	Sichuan	Tianjin	Xinjiang	Yunnan	Zhejiang
CMD	-0.65	-0.26	-4.89	-0.41	0.41	-0.51	-2.15	0.03	0.42	0.00	-7.32	0.00	-0.09	-3.14	-0.18
PNGE	-0.92	-0.01	0.00	0.55	0.00	1.58	-0.25	0.00	-1.42	0.00	-2.09	-0.13	-4.63	0.00	0.00
FMMD	0.10	0.02	-0.05	-0.36	0.47	0.00	-0.09	0.06	0.00	0.00	-0.08	0.70	-0.04	-0.53	0.00
NMMD	-0.02	0.00	0.22	-0.11	0.00	-0.33	-0.01	0.00	0.01	0.00	0.05	0.00	-0.01	-0.04	-0.02
FPS	0.71	0.00	0.01	-0.04	0.00	0.12	-0.07	0.00	0.03	-0.01	0.22	0.05	0.04	0.13	-0.01
FP	0.15	0.01	-0.03	-0.02	0.14	0.14	-0.09	0.00	0.03	0.00	0.06	0.18	0.00	0.06	-0.01
BP	0.15	0.00	0.00	-0.02	0.00	0.09	-0.01	0.00	0.01	-0.06	0.05	0.00	-0.01	0.07	-0.02
TP	0.00	0.00	0.00	0.00	0.00	0.00	0.00	0.00	0.00	0.00	0.00	0.00	0.00	-0.01	0.00
TI	0.14	-0.24	-0.03	-0.01	0.00	0.00	-0.12	0.00	0.00	-0.51	-0.32	-0.04	-0.03	0.00	-0.50
GOFP	0.02	-0.01	0.00	-0.01	0.00	-0.02	0.01	0.00	0.00	-0.07	0.00	-0.01	0.00	0.00	0.00
LFDRP	0.00	0.00	0.00	0.00	0.00	0.00	-0.01	0.00	0.00	0.02	0.01	0.00	0.00	0.00	-0.05

TPBCP	0.14	0.01	0.00	0.00	0.00	0.00	0.04	0.00	0.00	-0.01	0.02	0.00	-0.02	0.00	0.00
FM	0.01	0.00	0.00	0.00	0.00	0.00	0.00	0.00	0.00	0.00	0.00	0.00	0.00	0.00	0.01
PPP	-0.05	-0.12	0.03	-0.01	-0.19	0.00	-0.13	0.00	0.00	-0.03	-0.20	0.00	0.02	-0.01	0.02
PRMR	0.07	0.00	0.00	0.00	0.00	0.00	0.01	0.00	0.00	-0.10	0.03	0.00	0.00	0.00	0.00
CESA	0.00	0.02	0.00	0.00	0.00	0.02	0.02	0.00	0.00	0.00	0.02	0.04	0.00	0.00	0.00
PPC	-0.12	-0.07	-0.36	2.63	-0.24	0.84	1.08	-3.28	-5.22	-0.57	-0.81	-1.26	-4.18	-1.31	-0.11
RCMCP	-0.58	0.33	0.03	0.05	0.01	3.90	0.28	-0.21	0.35	0.22	0.62	-0.29	3.47	-0.45	0.43
MPP	0.53	0.00	0.00	0.03	0.00	0.06	0.03	0.00	0.00	0.11	0.09	-0.01	0.00	0.00	0.02
CF	0.02	0.01	0.01	0.04	0.00	0.00	0.00	0.00	0.00	0.00	0.00	0.00	-0.07	0.00	0.00
RPP	0.01	-0.02	0.00	0.00	0.00	0.00	0.02	0.00	0.00	-0.01	0.00	0.00	0.00	0.00	-0.06
NMP	15.24	0.12	0.33	-1.72	-0.33	2.08	-0.39	-0.05	0.55	-0.08	1.85	-0.30	0.44	2.86	0.00
SPFM	-9.97	1.00	-7.64	9.10	1.60	-3.38	-0.84	-0.94	-2.46	-6.31	-0.56	2.65	-8.77	-10.12	-0.77
SPNM	-0.68	-0.02	-0.14	0.13	-0.20	1.31	0.43	0.27	0.00	-0.34	0.00	0.06	0.77	-0.41	0.00
MP	0.21	0.01	0.00	-0.03	0.00	0.00	0.08	0.04	0.00	-0.06	0.01	0.04	0.02	0.00	0.02
OM	0.17	-0.20	0.01	-0.65	-0.13	0.01	-0.23	-0.03	-0.03	-0.04	-0.25	-0.02	0.00	-0.02	-0.04
ESP	0.12	0.00	0.00	-0.36	0.00	0.00	0.01	-0.03	-0.02	0.00	0.05	0.04	0.00	-0.01	-0.01
TE	2.75	-0.01	-0.04	0.14	0.00	0.00	-0.02	-0.03	-0.01	0.23	-0.03	0.00	0.00	0.01	0.08
EEM	-0.36	0.03	0.02	-0.06	0.00	0.20	-0.06	0.00	0.00	0.01	0.04	-0.01	0.00	0.00	0.03
ETE	-0.03	-0.01	0.00	-0.01	0.00	0.00	0.01	0.00	0.03	-0.03	0.01	0.04	0.00	0.00	0.03
IMCOM	0.00	0.00	0.00	0.00	0.00	0.00	0.01	0.00	0.00	0.00	0.00	0.00	0.00	0.00	-0.01
OMI	-0.01	0.00	0.00	-0.04	0.00	0.00	-0.12	-0.03	0.00	-0.01	-0.02	0.00	0.00	-0.04	-0.05
SW	0.00	0.00	0.00	-0.01	0.00	0.00	0.00	0.00	0.00	0.00	0.00	0.00	0.04	0.09	0.00
PSPH	-9.87	-17.27	-27.39	44.10	-7.93	4.37	-21.80	-4.96	-12.98	-19.77	-4.70	-27.56	52.67	-0.67	7.89
PSG	0.23	0.04	0.00	0.31	0.51	0.00	0.01	0.00	0.46	0.24	0.00	0.00	-0.04	0.00	0.00
PSTW	0.00	0.00	0.00	0.00	0.00	0.01	0.00	0.00	0.00	0.00	0.00	0.00	0.00	0.00	0.00

Table S3. Multi-period attribution analysis of energy intensity (Unit: %).

Sector	Anhui	Beijing	Chongqing	Fujian	Gansu	Guangdong	Guangxi	Guizhou	Hainan	Hebei	Henan	Heilongjiang	Hubei	Hunan	Inner Mongolia
CMD	2.15	0.00	3.50	-0.07	0.87	0.00	0.00	-6.95	0.00	0.76	-1.57	5.29	0.39	3.08	-0.88
PNGE	0.00	0.00	0.20	0.00	-0.27	0.10	0.00	0.00	0.00	0.15	0.63	-1.51	0.35	0.00	0.00
FMMD	-0.06	-12.52	0.00	-0.01	0.25	0.01	0.08	-0.10	-0.04	-0.14	-0.04	-0.04	-0.10	-0.06	-0.11
NMMD	-0.08	0.00	-0.19	-0.11	0.00	-0.04	0.18	0.00	0.00	-0.02	-0.05	-0.06	-0.24	-0.28	-0.03
FPS	0.01	-0.04	-0.03	-0.20	0.00	-0.15	3.47	0.00	0.00	-0.13	-0.21	1.62	-0.57	-0.24	-0.02
FP	-0.03	-0.01	-0.09	-0.26	-0.05	-0.10	-0.11	0.00	-0.12	-0.09	-0.20	0.17	-0.91	-0.16	0.06
BP	-0.01	-0.18	-0.03	-0.12	-0.02	-0.11	-0.10	-0.12	-0.01	-0.04	-0.14	0.59	-0.40	-0.09	-0.01
TP	0.00	0.00	0.00	0.00	0.00	0.00	0.00	-0.04	-0.01	0.00	0.00	-0.09	-0.01	0.01	0.00
TI	-0.04	0.00	-0.12	-0.68	0.00	-0.34	0.17	0.00	0.00	-0.06	-0.13	-0.32	-0.32	-0.08	-0.01
GOFP	0.00	0.00	0.00	-0.07	0.00	-0.28	0.00	0.00	0.00	-0.01	-0.01	-0.01	-0.10	0.00	0.00
LFDRP	0.00	0.00	0.00	-0.05	0.00	-0.08	0.01	0.00	0.00	-0.03	-0.03	0.00	0.00	-0.03	0.00
TPBCP	0.00	0.00	0.00	-0.05	0.00	0.00	0.17	0.00	0.00	-0.03	-0.02	-0.38	-0.06	-0.12	0.00
FM	0.00	0.01	0.00	0.00	0.00	-0.04	0.00	0.00	0.00	-0.02	0.00	-0.04	0.00	0.00	0.00
PPP	-0.17	0.00	-0.45	-0.58	-0.01	-0.46	0.88	-0.01	-0.11	-0.06	-0.22	-0.72	-0.55	-0.63	-0.01
PRMR	0.00	0.00	0.00	-0.01	0.00	-0.09	0.00	0.00	0.00	0.00	0.00	-0.01	0.00	0.01	0.02
CESA	0.00	0.00	0.00	-0.03	0.00	-0.20	0.00	0.00	0.00	0.00	0.00	-0.02	-0.01	0.00	0.00
PPC	0.06	0.35	0.12	-1.07	0.17	-0.19	1.10	-1.80	-1.59	-0.58	-4.25	-1.90	0.25	-0.93	-0.64
RCMCP	-0.16	-0.06	-0.68	-0.30	0.56	-0.03	0.25	-0.73	-1.39	-0.31	-0.55	-4.74	-4.37	-1.26	-0.13
MPP	0.00	-0.01	-0.03	0.00	0.00	-0.04	-0.02	0.00	-0.01	-0.01	-0.05	-1.19	-0.24	-0.15	0.00
CF	0.00	0.00	0.00	-0.01	-0.04	-0.01	0.00	0.00	0.00	0.00	-0.02	-0.33	-0.13	0.00	0.00
RPP	0.00	0.00	0.00	-0.04	0.00	-0.18	-0.02	0.00	0.00	-0.02	-0.02	-0.27	-0.13	0.00	0.00
NMP	-4.42	-1.10	-5.54	-4.25	-1.18	-2.52	4.28	-2.25	-0.37	-0.84	-1.76	2.16	-9.33	-5.07	2.20
SPFM	-4.71	-0.22	3.83	-8.41	1.97	-2.77	41.94	-4.75	0.49	-5.36	-7.99	-0.53	0.20	-7.97	-6.68
SPNM	-0.26	0.00	0.39	0.16	-2.52	-0.14	7.86	-0.65	0.00	-0.02	-1.57	-0.78	-0.54	-1.70	-0.56

MP	0.02	-0.01	-0.10	-0.09	-0.05	-0.33	-0.03	-0.65	0.05	-0.16	-0.16	-0.23	-0.21	-0.30	0.01
OM	-0.56	-0.01	0.02	-0.14	0.01	-0.19	-0.02	0.00	-0.02	-0.08	-0.32	-0.27	-0.33	-0.35	-0.01
ESP	0.03	-0.01	0.00	-0.02	0.01	-0.11	0.01	0.00	0.00	-0.07	-0.29	-0.26	-0.13	-0.24	0.01
TE	0.00	-0.45	-0.50	-0.07	0.00	-0.17	-0.06	-0.01	0.00	-0.10	-0.80	0.07	-0.80	0.00	0.00
EEM	0.01	0.00	0.01	-0.07	0.00	-0.40	0.01	0.00	0.00	-0.02	-0.10	-0.08	-0.03	-0.07	0.00
ETE	0.00	0.00	0.01	-0.04	0.00	-0.70	0.00	0.00	0.00	-0.01	-0.17	0.00	0.00	-0.08	0.00
IMCOM	0.00	0.00	0.00	0.00	0.00	0.00	0.00	0.00	0.00	0.00	0.00	0.00	0.00	0.00	0.00
OMI	0.00	-0.01	0.00	0.30	0.00	2.00	-0.02	0.00	0.00	0.00	-0.01	-0.14	-0.01	0.00	0.00
SW	-0.05	0.00	0.00	-0.01	0.08	0.00	0.00	0.00	0.00	0.00	0.00	0.00	0.00	0.00	0.00
PSPH	-10.59	-60.00	-31.84	-30.44	-28.72	-28.40	-69.44	-23.62	-8.73	-14.91	-20.52	11.77	-11.79	-26.31	-2.38
PSG	-0.03	-0.13	-0.04	-0.16	0.00	-0.28	-0.59	0.02	0.00	0.00	-0.12	0.20	0.00	0.00	0.07
PSTW	0.00	0.00	0.00	0.00	0.00	0.00	0.00	0.00	0.00	0.00	0.00	-0.02	0.00	0.00	0.00

Table S3 (continued)

Sector	Jilin	Jiangsu	Jiangxi	Liaoning	Ningxia	Qinghai	Shandong	Shanxi	Shaanxi	Shanghai	Sichuan	Tianjin	Xinjiang	Yunnan	Zhejiang
CMD	-4.06	-0.38	-0.86	0.48	-1.42	-0.23	-3.16	-0.85	7.60	0.00	-3.15	0.00	-0.42	3.55	0.13
PNGE	-0.19	-0.01	0.00	0.93	0.00	8.82	0.28	0.00	-1.65	0.00	1.77	-0.21	1.59	0.00	0.00
FMMD	-0.22	-0.03	-0.14	0.40	-0.38	0.00	-0.03	-0.12	0.00	0.00	-0.15	-0.86	-0.05	-0.40	0.00
NMMD	-0.02	-0.05	-0.70	-0.10	0.00	-0.25	-0.02	0.00	-0.03	0.00	-0.48	0.00	0.01	-0.20	-0.04
FPS	0.01	-0.04	-0.04	0.04	0.00	0.00	-0.28	-0.02	-0.41	-0.09	-0.73	-0.11	-0.10	-0.26	-0.04
FP	0.13	-0.03	-0.36	-0.01	-0.10	0.53	-0.15	-0.01	-0.67	-0.05	-0.22	-0.27	0.01	-0.08	-0.01
BP	0.29	-0.06	-0.09	-0.01	0.00	-0.49	-0.03	-0.02	-0.02	0.00	-0.62	-0.10	0.02	-0.07	-0.02
TP	0.00	0.00	-0.01	0.00	0.00	0.00	0.00	0.00	0.00	0.00	0.00	0.00	0.00	-0.06	0.00
TI	-0.04	-0.08	-0.05	-0.01	0.00	0.00	-0.34	-0.01	0.00	0.18	-0.30	-0.07	-0.01	-0.01	-0.18
GOFP	-0.02	-0.05	-0.04	-0.04	0.00	0.02	-0.05	0.00	0.00	0.05	0.00	-0.05	0.00	0.00	-0.07
LFDRP	0.00	0.00	-0.01	0.00	0.00	0.00	-0.01	0.00	0.00	-0.13	-0.08	0.00	0.00	0.00	-0.02

TPBCP	0.03	-0.07	0.00	-0.01	0.00	0.00	-0.14	0.00	0.00	0.00	-0.06	0.00	-0.02	0.00	0.00
FM	0.00	0.00	0.05	-0.01	0.00	0.00	-0.01	0.00	0.00	-0.01	-0.04	0.00	0.00	0.00	-0.03
PPP	0.12	-0.15	-0.23	-0.02	0.02	0.00	-0.28	-0.01	-0.05	-0.09	-0.48	-0.24	-0.06	-0.08	-0.30
PRMR	-0.10	0.00	0.00	0.00	0.00	-0.03	-0.02	0.00	0.00	-0.19	-0.06	0.06	0.00	0.00	0.00
CESA	0.01	-0.02	0.00	0.00	0.00	0.00	-0.03	0.00	0.00	0.00	-0.02	-0.07	0.00	0.00	0.00
PPC	0.44	-0.44	-0.48	0.96	-6.84	-1.54	-1.85	-1.24	10.89	-0.09	0.34	3.18	0.74	-2.22	0.36
RCMCP	-1.25	-1.05	-0.39	0.21	-0.05	0.43	-0.97	0.08	-0.73	-0.58	-2.26	-0.70	-1.26	-0.57	-0.62
MPP	-0.02	-0.01	-0.01	-0.04	0.00	-0.09	-0.05	0.00	0.00	-0.18	-0.23	-0.05	0.01	0.00	-0.04
CF	0.06	-0.08	-0.04	0.00	0.00	0.00	-0.03	0.00	0.00	-0.01	0.00	0.00	0.01	0.00	-0.07
RPP	-0.01	-0.01	0.00	-0.11	0.03	0.00	-0.06	0.00	0.00	-0.03	-0.01	0.00	0.00	0.00	-0.12
NMP	-8.48	-1.23	-3.16	1.49	-0.22	-2.84	-0.88	0.14	9.12	-0.16	-6.37	-0.19	-0.62	-1.62	-0.74
SPFM	0.08	-6.84	-9.37	10.55	-4.22	0.03	-5.31	-7.66	-0.48	0.31	-5.08	-26.27	1.30	-3.70	-1.89
SPNM	0.32	-0.05	-1.10	0.16	-0.33	3.70	-0.95	-0.08	-1.51	-3.20	-0.12	-0.15	0.81	-0.14	-0.07
MP	-0.09	-0.11	0.02	0.08	0.02	0.04	-0.14	-0.03	0.00	-0.11	-0.12	0.16	-0.02	-0.01	-0.13
OM	-0.08	-0.07	-0.08	-0.44	0.08	-0.03	-0.13	-1.07	-0.31	-0.22	-0.26	-0.09	0.00	0.00	-0.25
ESP	-0.01	-0.05	-0.03	0.44	0.00	0.00	-0.13	0.02	-0.24	-0.08	-0.34	0.04	0.00	0.00	-0.06
TE	-2.11	-0.08	-0.24	-0.19	0.00	0.00	-0.09	0.01	-0.35	-0.39	-0.25	0.00	0.00	-0.04	-0.31
EEM	0.23	-0.10	-0.05	0.08	0.00	-0.19	-0.17	0.00	-0.06	-0.10	-0.14	-0.04	0.00	0.00	-0.19
ETE	0.02	-0.02	0.00	0.01	0.00	0.00	-0.05	0.01	-0.06	0.01	-0.04	-0.11	0.07	0.00	-0.05
IMCOM	-0.02	0.00	0.00	0.00	0.00	0.00	-0.01	0.00	0.00	0.00	0.00	0.00	0.00	0.00	-0.01
OMI	0.01	0.00	0.01	0.06	0.00	0.00	0.08	-0.05	0.00	0.00	-0.03	0.00	0.00	-0.01	0.02
SW	0.00	0.00	0.00	0.00	0.00	0.00	0.00	0.00	0.00	-0.01	0.00	0.00	-0.02	-0.05	-0.01
PSPH	-41.71	-28.87	6.48	-17.89	10.50	17.13	-27.96	-2.32	-41.44	-18.11	-21.85	-29.91	-37.04	-40.37	-29.17
PSG	-0.22	-0.09	-0.01	-0.15	-0.24	0.00	-0.07	0.00	0.05	-0.44	-0.11	0.00	0.00	-0.12	0.00
PSTW	-0.02	0.00	0.02	-0.01	0.00	-0.06	-0.01	0.00	0.00	0.00	0.00	0.00	0.00	0.00	0.00
